# Female blue tits sing frequently: a sex comparison of occurrence, context, and structure of song

**DOI:** 10.1093/beheco/arac044

**Published:** 2022-06-20

**Authors:** Javier Sierro, Selvino R de Kort, Katharina Riebel, Ian R Hartley

**Affiliations:** Lancaster Environment Centre, Lancaster University, LEC Building, Lancaster, UK; Ecology and Environment Research Centre, Department of Natural Sciences, Manchester Metropolitan University, E437 John Dalton Building, Manchester Campus, Manchester, UK; Institute of Biology (IBL), Leiden University, Room number 7.4.17, Sylvius Building, Sylviusweg 72, Leiden 2333 BE, The Netherlands; Lancaster Environment Centre, Lancaster University, LEC Building, Lancaster, UK

**Keywords:** animal communication, bioacoustics, birdsong, *Cyanistes caeruleus*, female song, sexual characters

## Abstract

In species with mutual mate choice, we should expect adaptive signaling in both sexes. However, the role of female sexual signals is generally understudied. A case in point is female birdsong that has received considerably less attention than male song. This holds even for well-studied species such as the blue tit (*Cyanistes caeruleus*), an important model in evolutionary ecology. Although there have been anecdotal reports of female song from three populations, there are no quantitative studies on female song in this species. Here, we report systematic sampling from a population of individually marked blue tits over 3 years, revealing that females sang frequently throughout the sampling period. Notably, daytime singing of females occurred in functionally similar contexts as in males (agonistic, solo song, and alarm contexts) but females had lower song output than males and were not observed singing dawn song, while males showed long singing displays at dawn before copulations take place. Female and male song overlapped substantially in acoustic structure (i.e., same song types or peak frequency) but females had smaller individual song-type repertoires, shorter trills, and lower vocal consistency. Differential selection pressures related to functional differences in male and female song might explain the observed variation in acoustic structure. With the first quantitative study of female song in such a well-studied species, we hope to stimulate further investigations into the functions of female singing, especially in the Northern temperate zones where female song may have been overlooked, not only in this but perhaps in other monomorphic species.

## INTRODUCTION

Birdsong plays an important role in the acquisition of breeding resources, in mediating social conflicts and in mate attraction (Marler and Slabbekoorn 2004; Catchpole and Slater 2008) but also in pair coordination and in alarm situations (Cresswell 1994; Halkin 1997). Birdsong is therefore under sexual selection (but see Tobias et al. 2012) and has long been considered a predominantly male trait (Searcy and Andersson 1986; Collins 2004), a sex bias not unique to birdsong (Clutton-Brock 2009). Consequently, the function of birdsong has been studied mostly in males (Langmore et al. 1996; Riebel et al. 2005; Odom et al. 2014; Austin et al. 2021) despite reports of female song from the early days of modern birdsong research (Nice 1943; Robinson 1949; Nicolai 1959; Hoelzel 1986; Ritchison 1986).

It was not until this century that the first systematic worldwide survey was conducted, showing that female song is common in the basal clades of passerines, making concurrent male and female song the most likely ancestral state (Odom et al. 2014). With this shifting view, new questions arise regarding the function of female song and the selection pressures underlying sexual differences (Riebel et al. 2019). Even though the study of female song represents a very small fraction of the bird song literature (Odom and Benedict 2018), there is growing evidence, from an increasing number of species, that female song serves a variety of functions such as territory advertisement (Cooney and Cockburn 1995; Cain et al. 2015), mate attraction (Langmore et al. 1996), mate guarding (Reichard et al. 2018), or resource defense (Tobias and Seddon 2009) during inter- or intrasexual interactions (Krieg and Getty 2016; Kirschel et al. 2020). In non-duetting species, one of the most common functions of female song reported to date is related to the competition for breeding resources (and mates) between females (Langmore 1998; Austin et al. 2021). However, the documentation of female song is still too limited to make general statements, and more systematic research is needed to gain a complete picture of shared versus sex-specific functions of song in passerines (Riebel et al. 2019; Austin et al. 2021).

One unresolved question is why female song is much more common in the tropics and subtropics while it seems rare in the Passerida of the temperate zones (Slater and Mann 2004; Price et al. 2009; Odom et al. 2014). A current working hypothesis is that the presence of female song is the ancestral state in passerines, but that short breeding seasons, seasonal territoriality, and migration might be associated with the loss of female song (Benedict 2008; Price 2009; Odom et al. 2014). However, the evolutionary importance of female song seems to be underestimated, perhaps due to sampling biases (Garamszegi et al. 2007), for example by sexing a singing bird as male, especially in unmarked populations of monomorphic species (Eens and Pinxten 1998; Odom and Benedict 2018). To date, there have only been few systematic studies in Northern temperate regions that quantify female song and its functions (discussed in detail in Garamszegi et al. 2007; Riebel et al. 2019, but see for some notable exceptions Beletsky 1982; Baptista and Petrinovich 1986; Johnson and Kermott 1990; Hausberger and Black 1991; Baptista et al. 1993; Langmore et al. 1996; Yamaguchi 1998; Magoolagan and Sharp 2018; Wilkins et al. 2020; and Patchett et al. 2021; for descriptive studies of female song in Northern temperate regions). Importantly, by showing functional female song, these studies highlight the need to increase documentation and quantitative analyses of female song.

The blue tit, a well-studied passerine that breeds in the temperate regions of Europe and western Asia, might be a case in point. This songbird is a model species for studies of birdsong, mating systems, and other aspects of behavioral ecology (Mainwaring and Hartley 2019; Griffith et al. 2021). Even though blue tits show plumage dichromatism in the UV spectra (Andersson et al. 1998), males and females show only minor color and size differences to human observers, with much overlap in color intensity and size between the sexes (Cramp and Perrins 1993; Scott 1993). Female song in blue tits has been only reported anecdotally, but independently, in three different populations across Europe (Hinde 1952; Bijnens and Dhondt 1984; Mahr et al. 2016). Most surprisingly, there are no detailed, quantitative descriptions of female singing behavior, song structure, or context, despite the extensive literature of song research in this species (Stadler 1951; Hinde 1952; Latimer 1977; Doutrelant et al. 1998, 1999, 2000a, 2000b; Poesel and Kempenaers 2000; Poesel et al. 2001, 2004; Gorissen et al. 2002; Gorissen and Eens 2005; Poesel and Dabelsteen 2005).

In line with previous anecdotal reports (Hinde 1952; Bijnens and Dhondt 1984; Mahr et al. 2016), we encountered females singing during the collection of male song recordings and turned this into a systematic recording effort of both sexes in a population of individually marked birds. Based on these recordings, we here present the first quantitative analysis of the context, occurrence, and acoustic structure of female song in blue tits and compare it with the song of their male partners. Although there are some descriptive studies of female song in other bird species, these are predominantly from the tropics and subtropics. For temperate zone species, especially in Europe, studies involving systematic sampling of male and female singing within the same population and sampling scheme are uncommon, despite being a crucial step to develop testable hypothesis regarding female song functions (Riebel et al. 2019; Austin et al. 2021). Blue tits are of special interest in this context as their breeding biology is well studied in multiple long-term studies across Europe (Mainwaring and Hartley 2019). Blue tits are territorial and generally breed in monogamous pairs but are occasionally socially polygynous and frequently genetically polyandrous (Leech et al. 2001; Schlicht and Kempenaers 2021). Next to analyzing song structure, we will present analyses of the context of female singing, to test for associations with contexts for which female song has been reported, that is, solo singing, female–female competition, or alarm contexts (Langmore 1998; Mahr et al. 2016; Austin et al. 2021). Finally, we will also discuss the sexual similarities and differences in song structure in relation to possible functional differences based on the context in which we find females, and males, singing.

## METHODS

### Study species

All birds included in this study were part of a long-term monitored population (Mainwaring and Hartley 2009) breeding in 110 nest boxes (67 ± 1.15 broods per year in three breeding seasons), placed in deciduous and mixed woodland at Lancaster University campus, UK (54.01° N, 2.78° W). Nest boxes were made from 25-mm-thick softwood (hole 25 mm diameter, internal dimensions 125 × 125 × 200 mm), placed on tree trunks between 1 and 3 m above the ground and separated on average 25 m from each other (Supplementary Figure S1). Each year, adults were captured at the nest box using mist nets or trap-door traps at the entrance of their nest box, as well as at feeding stations with mist nets during the winter. Feeding stations were removed well before the start of the breeding season, to avoid impact of locally increased resources. Once caught, birds were measured (straightened, flattened, wing length to nearest mm, tarsus length with foot bent down, to nearest 0.1 mm, and head-bill length to nearest 0.1 mm), weighed (to nearest 0.1 g), and ringed with a unique combination of three colored rings and one numbered metal ring (Redfern and Clark 2001). Individuals caught during the breeding season are sexed in the hand based on the presence of a brood patch (females) or cloacal protuberance (males) (Svensson 1992). Birds were aged as first year or older than first year, based on plumage characteristics as described in Svensson (1992). All individuals included in this study were caught at least once during the breeding period.

### Sampling scheme and analysis of singing incidence

From the end of January to May 2018–2020, we conducted several walked transects per week to collect song recordings using a Marantz PMD661 recorder (48 kHz, 24-bit) and a Sennheiser ME67 microphone. The study was initially designed to investigate variation in song performance of males, but we regularly encountered singing females and concurrently established a data base of female song. We thus recorded males and females from the same population and season as recommended by Riebel et al. (2019). The linear transects followed the lines of nest boxes placed along strips of woodland (Supplementary Figure S1). When walking along a transect, we waited a maximum of 5 min at each territory (if no bird was detected) or until we had identified all blue tits detected, using binoculars to read the color combination of their leg rings. Only a fraction of the population was sampled each day (see last paragraph of this section); therefore, the starting points of daily transects would commence in the end point of the previous day’s transect, to cover the entire population equally. During all transects, whenever a blue tit was encountered singing, its behavior and song were recorded simultaneously. The observation ended whenever the focal bird stopped singing for more than 2 min or was out of sight. Once the ring combinations were identified for all birds detected, and after the song observation was recorded for singing birds, the observer continued to the next nest box along the transect. Sampling was predominantly blind with respect to the sex of the bird, as sexes look very similar at a distance in the field (Scott 1993). Thus, sex was determined by cross-checking the ring details with the database after the recording event. We used a dictaphone to take voice notes in a long, continuous recording that lasted the entire day of fieldwork, describing the entire singing observations as well as the ring details of the birds identified. The dictaphone also recorded the ambient sounds, including songs of blue tits that were nearby, and this was useful to record the first songs of a song bout if it was unpredictable, just before we started the high-quality audio recorder (for the operational definition of a song bout and song, see the “Analysis of song-type repertoire of female and male song” section). During the pre-breeding period, from January to the beginning of April, we made song recordings during daytime after sunrise throughout the morning until midday, since singing activity was limited at dawn during this period (Hinde 1952). In April and May, during the egg-laying (fertile) and incubation periods (mean first egg date was the 22nd April over the 3 years), dawn singing behavior was predictable (Hinde 1952), and thus we began sampling 1 h before sunrise until 1 h after sunrise. During the dawn chorus, when birds produce long singing displays starting 90–30 min before sunrise (Poesel et al. 2001; McGregor 2005; Gil and Llusia 2020), we did not conduct transects but waited in a position that was near a few neighboring territories to make song recordings from the local residential individuals each morning. This was because visibility was poor at dawn and, to confirm the identity of a singing bird, we kept following and recording the same bird until light levels were good enough for identification from the leg rings. The change in sampling scheme midway through the season was intended to optimize the collection of high-quality song recordings, based on previously reported seasonal changes in the timing of song in this species (Hinde 1952). Hence, our sampling scheme differed between the pre-breeding and breeding period, and it is thus less optimal to quantify seasonal changes in absolute song output. However, the recording of any singing individual encountered along the transects and around the nest boxes means that males and females were sampled the same way at every time point in the season. The data are thus suitable to compare male and female singing as both sexes were recorded in the same context, same population and with the same sampling schemes. From the data collected over 3 years, we created four data sets from the same group of females: one to compare song output, one to compare behavioral context of song, one to compare song-type repertoire, and one to compare acoustic structure in song between females and males. The same individuals could be part of one or all data sets. For a summary of the entire data base, see Supplementary Table S1.

### Analysis of song output

To provide a first description of female singing activity in blue tits, we calculated the probability of females singing as the proportion of females recorded singing per box visited, including all observations made during daytime before the breeding season (all observations before April), this is the song output data set. We then compared the probabilities of female and male singing per week using a Wilcoxon signed-rank tests, including a total of 29 weeks over 3 years, averaging 3.5 ± 1.8 days of sampling per week, 35.6 ± 22.9 boxes sampled per day, identifying a mean of 6.4 ± 5.6 females per day and 11.8 ± 6.9 males per day. Furthermore, we calculated the number of females and males recorded singing per minute in the field as well as measured the song output per observation, as the number of songs recorded per observation in 216 independent observations (see the sections “Analysis of behavioral context of female and male song” and “Analysis of song-type repertoire of female and male song” for operational definitions of observation and song, respectively). We compared the number of songs per observation between males and females with a Mann–Whitney *U* test, as data were not paired in this case. All measures are presented as mean ± 1 standard deviation, unless otherwise indicated.

### Analysis of behavioral context of female and male song

A posteriori, based on our field notes (dictaphone recordings), observations were categorized into one of four distinct behavioral contexts: alarming behavior, agonistic interactions, solo song, and dawn song (see Table 1 for operational definitions). For all except 10 cases, observations were classified into one of the four behavioral contexts. Occasionally, the behavioral context changed during a continuous focal observation. If this occurred, the observation was split into two separate, shorter, observations each assigned to the respective context (i.e., when switching from solo song to an alarm context). In some cases, two individuals could be recorded simultaneously (i.e., two breeding partners performing a joint alarm display) but only one observation was selected (randomly) in such cases, independently of sex. In some cases, alarm behavior may have been directed towards the human observer, suggesting these observations of male or female might have been observer induced, as has been reported by other authors (Bijnens and Dhondt 1984; Hinde 1952). However, assessing which alarm displays were or were not triggered by the observer is difficult based on our sampling scheme. In any case, as we observed both sexes engaging in alarm behavior and both sexes were exposed to a human observer during recordings, this context still allows for a comparison between sexes.To describe singing context in females, we measured the proportion of singing observations for each context, relative to the total number of observations within individual, including all observations along the 3 years of study. Then, for a sex comparison of singing context, we selected a subset of the recorded males in our population that were the breeding partner of, at least, one of the selected females during, at least, one breeding season. For this group of males, we used all observations recorded during the 3 years and, in each case, context was categorized with the same definitions as in females, using the voice notes recorded in the field during the transects. Breeding partners were defined as any two individuals that were observed provisioning the same brood, that is, nestlings in the same nest box. For this, we observed boxes directly to identify provisioning individuals from their leg rings, but also made video recordings of the entrance hole for at least 1 h, when the nestlings were 10–12 days old, with a Sony or Canon HD camcorder placed on a tripod 3–5 m away from the nest box.

**Table 1:** Operational definitions of behavioral and acoustic terms, by alphabetical order

Term	Definition
Agonistic interaction	Defined as any interaction where two or more blue tits produced conflict calls (Bijnens and Dhondt 1984) or actively chased, displaced, or attacked each other (Irschick et al. 2007) while singing. Observations were classified based on the highest level of aggression observed.
Alarm song context	Characterized by the production of song while 1) displaying mobbing behavior such as approaching closely and circling a potential predator (Cramp and Perrins 1993) or 2) producing song intercalated, or very close in time with high-pitched “tsee” calls or scolding calls. Both call types are typically produced in the presence of a predator (Hinde 1952; Latimer 1977; Bijnens and Dhondt 1984; Mahr et al. 2016)
Dawn song	A long vocal display that starts 90–30 min before sunrise (Poesel et al. 2001; McGregor 2005; Gil and Llusia 2020)
Bandwidth	Absolute difference between the maximum and minimum frequency of the note
Maximum and minimum frequency	Following Podos (1997), we measured the maximum and minimum frequency of each note in the mean power spectrum (window size: 1024 samples; amplitude threshold; −20 dB)
Peak frequency	The frequency with the maximum amplitude in the mean power spectrum (window size = 1024, window type = “Hanning”)
Solo songs	Characterized by an individual bird singing alone without any active social interactions with conspecific nor alarm responses
Song repertoire	Total number of different song types found across all recordings of a single individual during the entire study
Trill length	Duration of the trill in seconds, from the start of the first element until the end of last element.
Trill rate	Following Kirschel et al. (2009), trill rate was defined as the number of notes per second excluding the last rendition divided by the time between the start of the first note to the start of the last note in the trill (see “Male 1” in Figure 1). This is because all the notes in the trill include their corresponding internote gap except the last. Including all notes would cause a bias in trill rate due to the number of renditions in the trill.
Vocal consistency	Acoustic similarity between each note to the consecutive rendition within the trill using a spectrogram cross-correlation (SPCC) algorithm (Clark et al. 1987; Coleman et al. 2007; Botero et al. 2009). We calculated the maximum correlation of every pairwise SPCC with a maximum temporal offset of 20 ms and a temporal resolution of 1 ms. The spectrogram matrices were computed using an fast Fourier transform algorithm with a window size of 512 samples and 90% overlap between successive windows, “Hanning” window type
Vocal deviation	Using song of both sexes, we first calculated the upper bound regression between trill bandwidth and trill rate using bins of 100 Hz (Blackburn et al. 1992; Podos 1997). The vocal deviation is the orthogonal distance of each trill to the estimated upper bound limit (Supplementary Figure S2; see Podos 1997). The upper bound regression of vocal deviation including male and female song rendered a significantly negative slope (*r* (df = 35) = −0.90, *P* < 0.001; Supplementary Figure S2)

After excluding the observations made during the dawn chorus, as we had only observations for males in this context, the behavioral data set included 207 focal observations from 36 females and 29 males, after removing 9 observations with unknown behavioral context. For each individual, we estimated the proportion of observations per context in relation to the total number of observations for that individual, adding zeroes if an individual was never seen in a certain context. This led to a total of 195 data points to be used in the model, three for each of the 65 individuals. We fitted a binomial Generalized Linear Model or GLM (*glm* function from “stats” package; R Development Core Team 2016) using the proportion of observations for each individual per context as the response variable as a function of sex, behavioral context, and the full interaction between these two variables. Note such data structure implies only one data per individual per context (proportion), hence there are no repeated measurements and therefore no random terms were included. In this behavioral data set, we had different numbers of males and females because four male partners were never recorded, and six female–male pairs shared the same three males in same or different years. In many cases, we collected multiple observations of the same individuals on 2 or 3 different years (26/65) and in all except two cases, male–female partners were observed within the same season in at least 1 year. Only 3 out of 65 individuals were recorded during a year when breeding data were not collected for those 3 individuals.

### Analysis of song-type repertoire of female and male song

**Figure 1:**
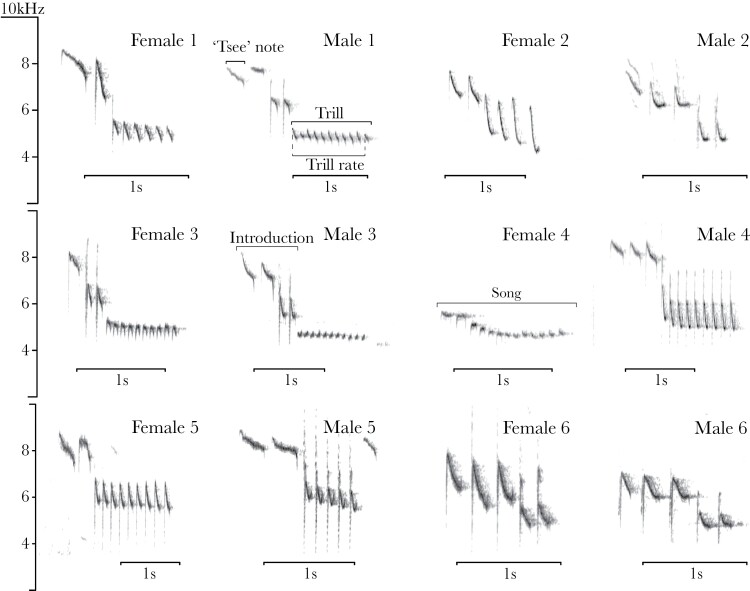
Spectrograms of female and male songs including most of the song types observed in females. For a complete repertoire of all females recorded in this study, please see Supplementary Figure S3. For each female, we selected the same, or similar, song type from her male partner. The basic terminology and structure of blue tit song are indicated in the spectrograms. The songs were selected to provide a good visual representation of song structure, not necessarily an example of statistical differences.

In the blue tit, as in many species, functional distinctions between songs and calls are not clear cut (Catchpole and Slater 2008). Often, blue tits integrate long bouts of calls in dawn song, a complex vocal display normally associated with song (Poesel et al. 2001, 2004). On the other hand, song is also used in contexts that are not strictly related with reproduction, such as alarm contexts (Mahr et al. 2016). For these reasons, we used structural criteria to define blue tit song based on spectrograms reported in previous studies (Bijnens and Dhondt 1984; Mahr et al. 2016). Song was defined as a vocalization composed of a few introductory, high-pitched notes followed by a trill, defined as the last part of the song where a note is repeated in succession (Figure 1) (Bijnens and Dhondt 1984; Cramp and Perrins 1993). Blue tit calls, which have a noisy rather than tonal acoustic structure (i.e., scolding or churring calls), were not analyzed. For the purpose of this study, we focused on song, including only those vocalizations that presented tonal structure to ensure that we were indeed describing female song rather than calls. This is a conservative approach since some blue tit song types include other types of sound (see B-syllables in Bijnens and Dhondt 1984). For our description of song and for the song analyses, a note was defined as a continuous trace in the spectrogram, separated from other notes by silent gaps longer than 10 ms. Each recorded individual sang several stereotyped song structures, referred to as song types, and these are comparable between individuals within a population (Figure 1). During singing, blue tits repeat the same song type many times, alternated with silent pauses (i.e., discontinuous singers), before switching to a different song type which results in so-called song type bouts (Poesel et al. 2004). Each of these renditions are referred to as a song (Figure 1) and one song was sufficient to count a bird as singing during one observation.

Blue tits have small individual song-type repertoires that range from three to eight different types (Bijnens and Dhondt 1984). Since blue tits repeat the same song many times before switching, repertoire size is often estimated on the basis of the long, sustained singing displayed during the dawn chorus (Doutrelant et al. 2000a; Poesel et al. 2004). We did not record cases of females singing dawn song, and female daytime singing, like that of males, consisted of much shorter singing bouts. Hence, to compare repertoire usage between sexes, we counted the number of distinct song types across all recording days of the same individual made during daytime singing. Blue tits are generally assumed to be close-ended learners (Bijnens 1988; see also Hansen et al. 2008; for imprinted species recognition of song) and based on this assumption, we grouped recordings across years for those individuals recorded in multiple seasons (see Bijnens and Dhondt 1984 for similar methodology). Hence, we created a subset, hereafter the repertoire data set, selecting those females recorded on two or more dates with more than 10 songs recorded overall, including 19 females.

We categorized song types by visual inspection of spectrograms (Bijnens and Dhondt 1984; Doutrelant et al. 1998) in Audacity (Mazzoni and Dannenberg 2014) (window type: “Hanning,” window length 1024 samples, 90% overlap and −80 dB range). Based on the song delivery mode of blue tits, that repeat the same song type many times, we focused on the switching points between song types within individuals as the main criteria for identifying song types. The switching point between song types was easily identifiable even if song types were similar. Some of the main features of song that further helped in categorizing song types were trill rate, frequency modulation of trill notes, the trill length, and the structure of the introductory part (Figure 1). But note that, in all cases, the categorization was based on visual inspection of spectrograms. For each of the 19 females, we selected her male partner to compare song-type repertoires between sexes. In almost all cases, we collected a larger sample of song recordings for the male than for the female (see Supplementary Table S1). From 38 individuals, 21 were recorded in more than 1 year (10 females and 11 males) and, within pairs, male and female partners were always recorded within the same season in at least 1 year. For each recording, we transformed date into Julian date, taking the first of January of each year as origin resulting in a standardize date across seasons. Within each female–male pair, we used the dates of the female recordings to select the nearest dates of recording for male songs, regardless of the year of recording. By doing this, we reduced potential seasonal variation due to singing modes. After matching female and male recording dates, we selected the same number of songs from each male recording unless there were fewer male songs than female songs for that match. The count of songs started always in the first song recorded. In this case, we also excluded male dawn chorus recordings to avoid a contextual bias on the comparison of song repertoires between sexes. While this method means that we might not have included the complete repertoire of each individual, it allowed us to compare repertoire usage between the sexes. We used Wilcoxon signed-rank tests to compare the number of song types of females and males within pairs.

### Analysis of acoustic structure

For detailed analysis of acoustic parameters of female song, we chose a subset of females with song recordings of high signal-to-noise ratio, hereafter referred to as the acoustic data set. We then selected individual males that were breeding partners of these females in at least one breeding season. Similar to the male sample in the analysis of song-type repertoires, we selected the same number of songs for each male partner, recorded on similar dates and excluding song recorded during the dawn chorus. This resulted in a data set of 786 high-quality recording songs, 435 songs from 32 females (14 ± 12 songs per individual) and 351 songs from 28 male partners (12 ± 13 songs per individual). For 2 of 32 females, the male partner was not recorded at all. Four female–male pairs shared the same two males in the same or different years. From 30 females that were matched with a male partner, 7 were recorded in different years than their mate. In 23 pairs, both partners were recorded, at least once in the season they were breeding together. Finally, 31 of 32 females were recorded singing during the same year when they were recorded breeding. From all 60 individuals, we had the exact date of first egg for 90 nests over three seasons to calculate the “weeks to first egg” of each recording. In eight cases across 3 years, we did not have breeding data hence, as an estimate of the weeks in relation of first egg of each recording, we used the mean week of first egg for the entire population in that year. Since all females laid their eggs within 3 weeks of the year (weeks 15–17), the possible error introduced should not impact our analysis.

We conducted all acoustic analysis using Audacity and R software (package “tuneR”: Ligges 2013; package “seewave”: R Development Core Team 2016; Sueur et al. 2006). For each individual, we analyzed a maximum of 10 songs for each song type and each date recorded. Acoustic measurements were made only in the trill since the introductory notes of blue tit song are more variable and may be absent in some song types. Despite having selected high-quality recordings, we still had to exclude some notes that were masked by extraneous sounds but included the rest of the song in the analysis (7.04 ± 5.3% of notes in females and 6.4 ± 5.5% of notes in males). We did this to avoid biasing the sample towards shorter songs, as longer songs were more likely to be partly masked.

Every note was manually labeled in the spectrogram, using the cursor to mark the start and end times in Audacity, and these time marks were exported as a text file. With this file, we used R software to cut out each note from the recording and save it as a single, normalized *wav* file. Following Podos (1997), we measured the peak, maximum, and minimum frequency of each note and from this we derived the bandwidth of the note (Table 1). We also took measurements of song performance including vocal consistency (sensu de Kort et al. 2009), trill length, trill rate (sensu Kirschel et al. 2009), and vocal deviation (sensu Podos 1997). Table 1 shows detailed operational definitions of each acoustic variable. All acoustic variables were measured in each note and then, a mean value per song was calculated for the statistical analysis.

For the acoustic analysis, we built five Linear Mixed-Effects Models (*lmer* function from “lme4” package; Bates 2010) to investigate sex differences for each of the following five parameters: peak frequency, vocal consistency, trill length, trill rate, and vocal deviation (Table 3). Note that sample sizes varied slightly between models. For instance, measuring trill rate unbiased (sensu Kirschel et al. 2009) is only possible in trills of at least three notes, which also affects the measurement of vocal deviation. In some trills, recording quality was insufficient to take spectral measurements but trill rate or trill length were easily measured. In other cases, signal-to-noise ratio was sufficiently high to measure peak frequency, but vocal consistency was not measured due to extraneous sounds in the background outside the spectral range of the note. For each model, the exact sample size is specified in Table 3. To describe the spectral features of song, we selected only the mean peak frequency of each song, because it is a robust measurement that is little affected by recording quality (Linhart et al. 2012) and it was strongly correlated with the maximum frequency (*r* = 0.90, *P* < 0.001, degrees of freedom [df] = 759) and the minimum frequency (*r* = 0. 89, *P* < 0.001, df = 759). As explanatory variables we used sex (male and female), age (first year or older than first year), and weeks in relation to first egg date to account for seasonal variation (week of first egg = 0) (sensu Schlicht and Kempenaers 2020). We included sex-specific interactions with age and season effects (in weeks in relation to first egg) to investigate their potential effects on each sex specifically. To model peak frequency, we also included the tarsus length and its interaction with sex, since this song feature could be affected by sexual dimorphism in body size. To account for repeated measurements, observations were nested within individual and within pair, using both variables as random effects.

**Table 3:** Output of the full-average model for each song trait, comparing male and female song in five LMMs with a Gaussian family distribution and an identity link function

	Fixed effects estimates						Random effects estimates			
Parameter	Variable	Estimate	2.5% CI	97.5% CI	*Z* value	Relative importance	Variable	Variance	*R* ^2^_*m*_	*R* ^2^_*c*_
Peak frequency (kHz) *N* = 761 songs, 60 individuals	Intercept (female, yearling)	5.02	4.838	5.203	54.01	—	Individual	0.15 (0.08–0.24)	0.12	0.692
	Age (yearling vs. older)	0.152	0.075	0.229	3.854	1	Pair	0.02 (0.00–0.10)		
	Weeks to first egg	−0.255	−0.375	−0.134	4.145	1	Residual	0.09 (0.08–0.10)		
	Sex (female vs. male)	0.025	−0.231	0.28	0.191	1				
	Age: sex (female–yearling vs. older vs. male–yearling vs. older)	−0.232	−0.325	−0.14	4.93	1				
	Weeks to first egg: sex (female–season vs. male–season)	0.073	0.027	0.272	0.843	0.489				
Vocal consistency (spectrogram cross-correlation algorithm score) *N* = 745 songs, 60 individuals	Intercept (female, yearling)	0.867	0.84	0.895	60.811	—	Individual	0.002 (0.001–0.003)	0.125	0.435
	Weeks to first egg	0.037	0.025	0.049	6.234	1	Pair	0.00 (0.00–0.001)		
	Sex (female vs. male)	0.024	0.016	0.07	1.01	0.556	Residual	0.004 (0.003–0.004)		
Trill length (s) *N* = 786 songs, 60 individuals	Intercept	0.483	0.402	0.564	11.728	—	Individual	0.06 (0.03–0.08)	0.105	0.609
	Sex (female vs. male)	0.196	0.078	0.315	3.284	—	Pair	0.00 (0.00–0.02)		
							Residual	0.04 (0.04–0.05)		
Trill rate (notes/s) *N* = 571 songs, 58 individuals (31 females, 27 males)	Intercept (female, yearling)	8.582	7.998	9.166	28.82	—	Individual	2.9 (1.6–4.7)	0.041	0.744
	Age (yearling vs. older)	0.200	−0.09	0.871	0.763	0.513	Pair	0.48 (0.00–2.1)		
	Sex (female vs. male)	−0.075	−1.089	0.721	0.243	0.405	Residual	1.2 (1.07–1.4)		
	Age: sex (female–yearling vs. older vs. male–yearling vs. older)	−0.127	−0.939	−0.054	0.519	0.256				
Vocal deviation (orthogonal distance) *N* = 550 songs, 58 individuals (31 females, 27 males)	Intercept (female, yearling)	5.963	4.948	6.978	11.517	—	Individual	3.2 (0.80–6.5)	0.023	0.317
	Sex (female vs. male)	0.321	−0.941	1.985	0.503	0.615	Pair	1.1 (0.0–4.2)		
	Weeks to first egg	−0.034	−1.179	1.007	0.096	0.39	Residual	9.9 (8.7–11.2)		
	Weeks to first egg: sex (female–season vs. male–season)	−0.162	−2.345	0.477	0.35	0.174				
	Age (yearling vs. older)	0.06	−0.126	0.719	0.389	0.201				

Variance attributed to individual identity (ID) and pair identity (Pair ID), fitted as a random effects, as well as residual variance (Residual) is shown by the standard deviation with the associated 95% CI. For each fixed factor, we present the model estimate, the 95% CI around the estimate, the *Z* statistic derived from Wald tests, and the relative importance of that factor in the final model. The relative importance reflects the number of models, from the subset selected (ΔAICc < 2) that included that specific variable. The last two columns show the marginal *R* squared represented as *R*^2^_*m*_, as the variance explained only the fixed terms, and the conditional *R*-squared represented as *R*^2^_*c*_, as the variance explained by both fixed and random terms. For each fixed effect, the specific levels to be compared are shown in parenthesis.

### Statistical analysis

Statistical analyses were carried out in R software (R Development Core Team 2016). To validate all models, we confirmed that the residuals were homoscedastic and showed a normal distribution using diagnostic plots (Zuur et al. 2009; Knief and Forstmeier 2021). We also tested for potential multicollinearity among the explanatory variables of the model by visual inspection of paired correlation plots and by estimating the Variance Inflation Factor (VIF) (*vif* function from “car” package; Fox and Weisberg 2019) for each variable with and without the interactions. Multicollinearity among explanatory variables was assumed if VIF was greater than 3, excluding those variables that were the product (interaction) of simple variables (Zuur et al. 2009). To find which factors were important in explaining variation in song, we used an information theoretic approach, computing all possible model combinations and ranking them using the Akaike Information Criterion for small samples (AICc, *dredge* function in the package “MuMIn”; Barton 2011). This procedure compares the fit of all possible models while penalizing model complexity, in terms of the number of explanatory variables included. We selected all models that had ΔAICc <2, in relation to the model with the lowest AICc score (best model), to compute the full-average model as the final model (Burnham and Anderson 2002; Burnham et al. 2011). We used the relative importance of each factor in the final model together with the coefficients and estimated confidence intervals (CIs) with a threshold of 95% (Nakagawa and Cuthill 2007; Burnham et al. 2011), concluding there was a significant effect if the CI did not overlap with zero. In the process of model selection, some predictors were dropped from the final model, indicating that their impact on the response variable was low. Finally, we calculated the *R*^2^ or *R*^2^_GLMM_ (r.*squaredGLMM* from “MuMIn” package) of the full models to measure the goodness of fit (Nakagawa and Schielzeth 2013). All numerical variables are scaled and centered so model estimates are standardized (Gelman 2008). Other packages used in data management and data visualization during the analysis were “stringr” (Wickham and Wickham 2019), “plyr” (Wickham and Wickham 2020), “data.table” (Dowle and Srinivasan 2021), “ggplot2” (Wickham 2016), “geosphere” (Hijmans 2019), and “lattice” (Deepayan 2008).

## RESULTS

In 3 years, we recorded 101 singing observations from 36 different females (Supplementary Table S1). Each year, we identified 49% ± 18% of breeding females during field sampling hours (39/68 in 2018, 42/69 in 2019, and 10/68 in 2020). Of those identified females, we recorded 47% ± 14% females singing (14/39 in 2018, 18/42 in 2019, and 12/19 in 2020). Of all 101 observations, 70 observations on 28 individual females were collected from January to April, during daytime transects, the remaining 31 observations (16 individuals) were collected in April and May, when sampling was mostly focused on dawn chorus (until 1 h after sunrise), although females were not observed producing dawn song.

### Song output

During the daytime transects from January to April, we recorded a female singing every 110 min in the field, at 8.2% ± 6.6% of the boxes visited. For the same period and sampling scheme, we recorded a male singing every 33 min of fieldwork, at 28.2 ± 21.5% of the boxes visited, which is significantly higher than females (*W* = 0, *P* < 0.001, 5% CI = −0.23, 95% CI = −0.12). Per observation, we found that females’ song output (10.2 ± 10.6 songs per observation) was significantly lower than males’ song output (24.3 ± 23.5 songs per observation, *W* = 3642.5, *P* < 0.001, 5% CI = −11.0, 95% CI = −5.00). Note that observations after 1st of April were not included in these analyses, as the sampling scheme changed.

### Behavioral context of song

**Figure 2:**
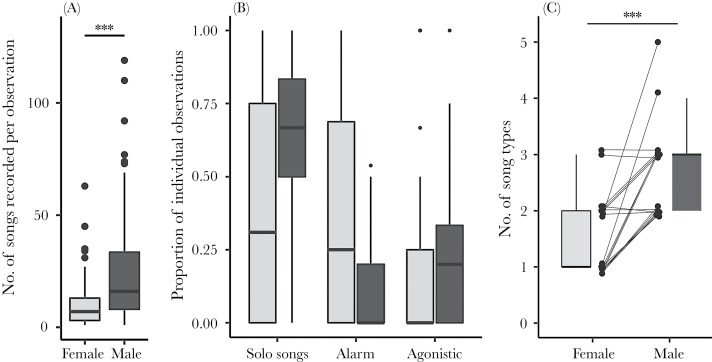
Sex differences in song output (a), singing context (b), and in song-type repertoires (c). Panel (c) shows the comparative analysis of song-type repertoire between sexes using a box and whiskers plot at each side while the points (raw data) are shown in the center linking male–female pairs with lines. Significance code: ***P < 0.001 (Mann–Whitney *U* test in (a) and Wilcoxon signed-rank test in (c)). Box and whiskers plot show median, the 25% and 75% quartiles as lower and upper hinges, respectively, and the whiskers stretch to the maximum (and minimum) value within 1.5 times the interquartile range. Points outside this range are shown individually.

For 36 females and 29 males, we conducted the behavioral analysis of song context. In this model, there was no collinearity among the explanatory variables. We found that females sang significantly less often during agonistic than during solo song, while no differences were found between solo song and alarm context (Table 2, Figure 2a). Males sang proportionally more often during solo song than females (see estimate for “sex” in Table 2, Figure 2a). Females sang more often in the alarm context, relative to solo song, compared to males (see estimate for interactions “sex: alarm” in Table 2, Figure 2a). There was no difference in the use of song during agonistic interactions, compared to solo song, between the sexes (see estimate for interactions “sex: agonistic” in Table 2, Figure 2a).

**Table 2:** Behavioral context of song compared between sexes by fitting a GLM binomial model using a log link function

	Log-transformed model estimates			Back-transformed model estimates			
Fixed effect	Estimate	2.5% CI	97.5% CI	Estimate	2.5% CI	97.5% CI	*Z* value
Intercept (female, solo song)	−0.351	−0.773	0.060	0.704	0.462	1.062	−1.66
Alarm (female–alarm vs. solo)	0.000	−0.588	0.588	1.000	0.555	1.80	0.00
Agonistic (female–agonistic vs. solo)	−1.207	−1.908	−0.541	0.299	0.148	0.582	−3.476
Sex (female–solo vs. male–solo)	0.867	0.310	1.434	2.38	1.364	4.197	3.028
Sex: alarm (female–alarm vs. solo vs. male–alarm vs. solo)	−2.135	−3.001	−1.29	0.118	0.05	0.275	−4.901
Sex: agonistic (female–agonistic vs. solo vs. male–agonistic vs. solo)	−0.642	−1.538	0.264	0.526	0.215	1.302	−1.399

For each fixed factor, we present the model estimate, the 95% CI around the estimate, and the *T* statistic, both the log-transformed as well as the back-transformed estimates in the original scale. The *R*^2^ for the full model was 0.327. After model selection, only the full model was selected, as the second-best model had a ΔAICc greater than 2. Therefore, the relative importance is not calculated. The model takes the female category of sex variable as well as the solo song category from the context variable as reference levels (included in the intercept). For each fixed effect, the specific levels to be compared are shown in parenthesis.

From 42 agonistic observations with song, 15 were from females. From these, in three cases, a female approached and sang while her partner interacted agonistically with another individual (twice with birds of unknown sex and once with another male). Females showed agonistic behavior (conflict calls, chase, displacement, or attack) in the other 12 female agonistic observations, 3 during a contest between two male–female pairs, 5 during a contest with another individual of unknown sex, and 4 during a contest with another known female. In these four cases of female–female interactions, we observed two cases of direct physical aggression (attack). In two cases of female active agonistic interactions, their male partner approached and sang but did not contribute physically to the interaction. In 2 of 27 male agonistic observations selected for this study, we observed direct physical aggression to an opponent of unknown sex.

### Song-type repertoire

Visual inspections of the spectrograms of all songs recorded in the study (1113 female songs and 1087 male songs) revealed that males and females used the same song type categories (printed spectrograms of the full individual song-type repertoire for 36 females can be found in Supplementary Figure S3). Considering the relative dates within season, male and female partners were recorded a mean of 3.0 ± 21.4 days apart and the difference in the total number of songs recorded within pairs was 1.4 ± 13.4, from a total of 2200 songs analyzed for both sexes combined. Comparing 19 females (4.1 ± 1.8 dates and 59 ± 45 songs) directly with their 19 male partners (3.6 ± 1.8 dates and 57 ± 42 songs), we found that females used significantly fewer song types than males (females = 1.0 ± 1.0 vs. males = 3.0 ± 1.0 song types, median ± interquartile range; *N* = 19 pairs, *W* = 57.5, *P* < 0.001, 5% CI = −1.00, 95% CI = −2.00; Figure 2b). For 13 of the 19 females, we only recorded one song type, and this included three of the most recorded females (recorded on at least four different dates and 93 ± 46 songs sampled; see Supplementary Table S1). In contrast, none of the males in our population sang fewer than two song types.

### Acoustic structure of song

**Figure 3:**
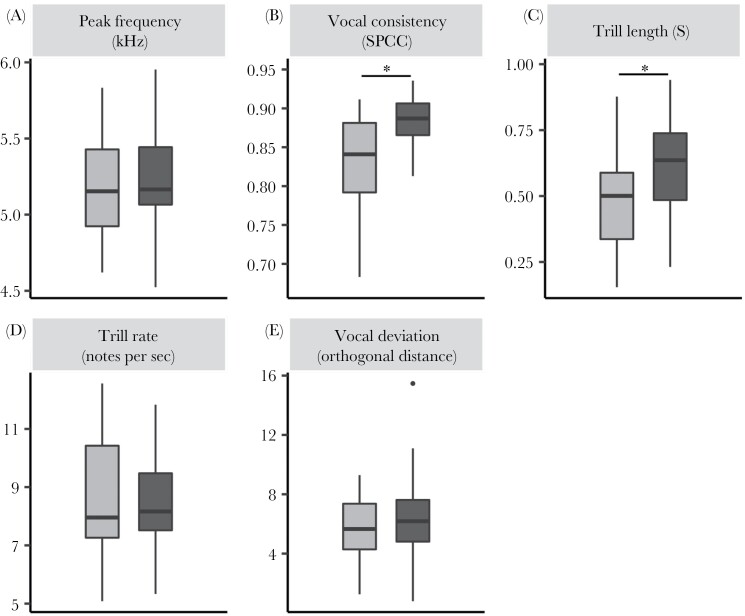
Comparison of female and male acoustic variables (females in gray and males in black) using box and whiskers plots, built as in Figure 2. Females sing with lower vocal consistency (b) and shorter trill length (c) than males. Peak frequency (a), trill rate (d), and vocal deviation (e) were not statistically different between sexes. The asterisk symbol means that the CI for the sex estimate in those models does not overlap with zero.

**Figure 4:**
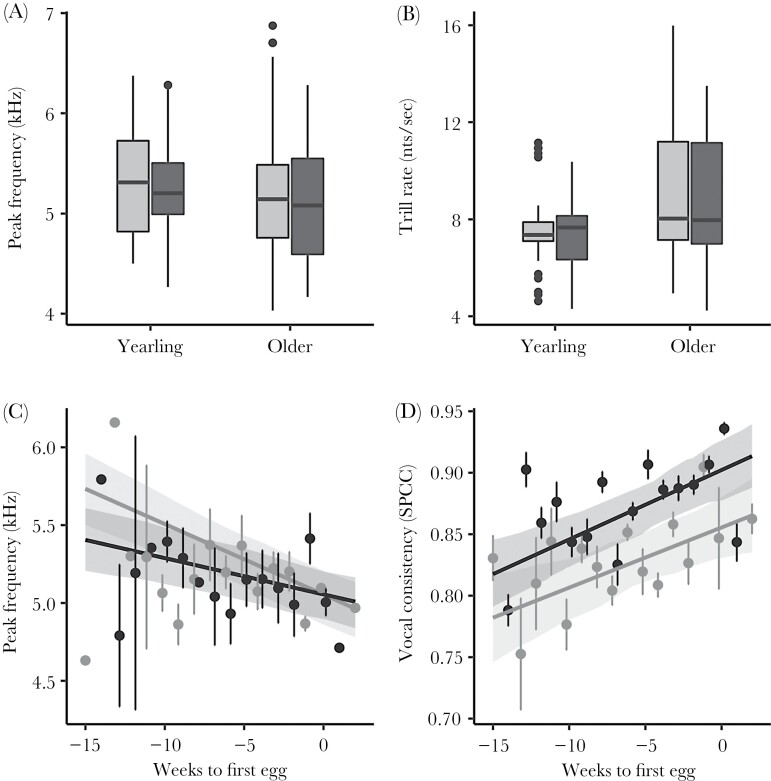
Significant correlations of age with peak frequency (a) and with trill rate (b) as well as season with peak frequency (c) and vocal consistency (d). Points represent raw data and lines represent predicted values from the model (Table 3), with the associated CI in the shaded area around lines. Females are represented in gray and males in black.

**Figure 5:**
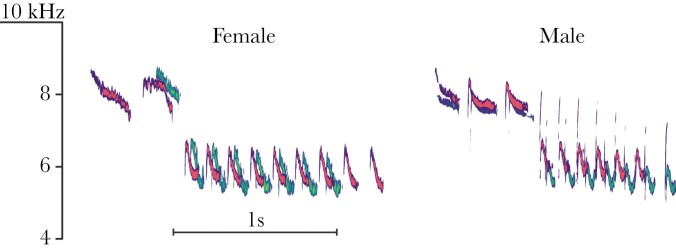
Spectrograms showing examples of spectral flexibility along the season within individuals. On the left, the same song type recorded from the same individual female, 6 weeks before laying the first egg (winter, in red) and 2 weeks before the first egg (spring, in green). Similarly, a male example is shown from 10 weeks before first egg date (winter, in red) and during the week of first egg (spring, in green).

We found no collinearity among the explanatory variables in these models. Female blue tits sang with significantly lower vocal consistency and produced shorter trills than males, but they did not differ in peak frequency, trill rate, or vocal deviation (Figure 3, Table 3). In both males and females, vocal consistency increased, and peak frequency decreased, from winter towards spring (fertile period), although males’ seasonal decrease in peak frequency was significantly lower (Figures 4 and 5, Supplementary Figure S4, Table 3). In both males and females, older birds showed significantly lower peak frequency than first-year birds, although such change was significantly smaller in females than in males (Figure 4, Table 3). In the final model, we found a significant increase in trill rate with age in males, but not in females (Table 3), even though the increase was significant in both sexes in the full model (Supplementary Table S2). Contrary to expectation, we did not find a significant correlation between peak frequency and tarsus length. As all models included the full interaction of sex with the other predictors, all the effects of age, season, and tarsus length were estimated specifically for each sex within the same model, although model estimates for such interactions must be taken with care given our sample size. The estimates and the associated CIs for all parameters in the full models are given in Supplementary Table S2. Supplementary Tables S3 and S4 show model selection tables, including all model combinations with an ΔAICc lower than 7 with respect to the best model for all song traits, although only the models with ΔAICc <2 were selected to build the final model.

## DISCUSSION

This first season-long, transect-based quantification of female song in blue tits showed that female blue tits sing regularly throughout the season across a variety of behavioral contexts. These include alarming behavior, solo singing, and agonistic contexts, although females show lower song output in both rate of singing and number of songs and were not observed to produce dawn song. The structural analysis of song showed that females used similar song types as males, overlapping substantially in song parameters, but also showed quantitative differences. Females had smaller song-type repertoires and sang shorter trills of lower vocal consistency. Our results also suggest that female song traits have communicative value as we found that female song 1) changes seasonally, as peak frequency decreased and vocal consistency increased towards the fertile period, and 2) correlates with age class, as older females had lower peak frequency. The same relationships were also found in male song.

Throughout the season, female song was frequent and far from a rare phenomenon in our study population. As birdsong is a complex behavior that involves specialized neural centers, muscle structures, and developmental learning processes (Suthers 2004), it seems unlikely that such behavioral and physiological adaptations are specific to our population. Nevertheless, large-scale social and environmental factors have been shown to influence female song across species (Benedict 2008; Price et al. 2009) and it is possible there are also other environmental factors that may modulate singing rates within species, across populations. For instance, higher habitat fragmentation due to an urbanized environment in our population may increase female–female competition for breeding opportunities (Bain et al. 2014) which may in turn raise singing rates in females (Kluyver 1955; Langmore and Davies 1997). This hypothesis could be tested in the future by investigating and comparing additional populations. Regardless of potential population differences in female singing rates, our observations put previous anecdotal reports of female song from three distinct populations into a new perspective (Hinde 1952; Bijnens and Dhondt 1984; Mahr et al. 2016; see also Gorissen and Eens 2005; for a report of female “subsong” inside the nest). This combined evidence suggests that female song is part of blue tit vocal signaling, a proposition worth investigating across other populations in Europe.

The blue tit can thus be added to a growing list of monomorphic European bird species with prolific female singing (for a review of European species with female song, see Garamszegi et al. 2007), despite the overall lower incidence of female song of Passerida in the Northern temperate zones compared with other biogeographic regions (Garamszegi et al. 2007; Odom et al. 2014; Webb et al. 2016). Moreover, the regular incidence of female song in the blue tit is in line with the results of worldwide surveys showing a higher incidence of female birdsong in non-migratory species (but see Nilsson et al. 2008) with low sexual dichromatism (Webb et al. 2016) that hold year-round territories (Benedict 2008; Price 2009; Logue and Hall 2014; Odom et al. 2014; Riebel et al. 2019). As song is often used to sex singing individuals in unmarked populations, and female song appears more common in monomorphic species, the incidence of female song might be underestimated (Riebel et al. 2005; Odom et al. 2014; Odom and Benedict 2018). It seems possible that there are other European species, like the blue tit, where female song might have been misclassified as incidental or absent.

Another reason that could explain why female song has been overlooked in blue tits is the fact that most studies collect (male) song recordings during the dawn chorus (Doutrelant et al. 1998, 2000a; Poesel and Kempenaers 2000; Poesel et al. 2001, 2004; Gorissen et al. 2002). In our population, we did not observe females producing such long, sustained singing at dawn and, if this pattern holds for other populations, it could explain why female song was so long overlooked in this intensively studied songbird species. In line with this, it is worth noting that the few studies that anecdotally reported female blue tit song in their populations recorded song also during daytime singing throughout the season, not only in the dawn chorus (Hinde 1952; Bijnens and Dhondt 1984; Mahr et al. 2016)

The regular occurrence of female song and its structural similarity to male song raises questions about its potential function(s). Although our study was purely observational, the contexts, in which we observed female song, allow to develop some working hypotheses regarding its function(s). One of the common contexts in which female blue tits sang was alarming behavior. This supports the findings by Mahr et al. (2016) that showed that blue tits sing upon presentation of simulated predator (a taxidermy mount of a sparrowhawk, *Accipiter nisus*). Blue tits are thus among a growing number of species known to produce song in the presence of predators (Langmore and Mulder 1992; Cresswell 1994; Laiolo et al. 2004), suggesting song as a potential antipredator strategy. The other two frequent contexts of female song were agonistic interactions and solo singing, which could indicate a function for female song in territory defense or mate attraction (Langmore 1998; Mikula et al. 2020; Austin et al. 2021). A territorial function of female song has been demonstrated in several species using playback experiments (Hoelzel 1986; Cooney and Cockburn 1995; Krieg and Getty 2016; Magoolagan and Sharp 2018). The observation that both sexes use the same song types could facilitate intersexual interactions in such agonistic context, since song type matching is an aggressive signal in this and related species (Krebs et al. 1981; Langemann et al. 2000; Poesel and Dabelsteen 2005). A shared repertoire of song types also suggests that both sexes learn from the same models in the same locations (Riebel 2003).

More specifically, female song could serve a function during intrasexual competition, as we observed females singing during aggressive interactions with other females. While the sex of the opponent was unknown in some agonistic interactions, we never saw a confirmed agonistic interaction between a single female and a male. A similar role of female song during intrasexual conflicts has been suggested in other species. In dunnocks (*Prunella modularis*) and great reed warblers (*Acrocephalus arundinaceus*), artificially high female–female competition increased the incidence of female song during intrasexual conflicts (Kluyver 1955; Langmore and Davies 1997). Female European starlings (*Sturnus vulgaris*) and dark-eyed juncos (*Junco hyemalis*) produced song during aggression directed towards caged females placed in their territory (Sandell and Smith 1997; Reichard et al. 2018). Female song in duetting Peruvian antbirds (*Hipocnemis peruviana*) could have evolved as a signal jamming strategy, potentially disrupting extra-pair mate attraction by the male (Tobias and Seddon 2009), as has also been suggested for female barn swallows (*Hirundo rustica*) (Wilkins et al. 2020). In eastern whipbirds (*Psophodes olivaceus*), females approached more closely to playback of female solo song than to male or duet songs (Rogers et al. 2007). Like blue tits, many of these species are opportunistically polygynous (Langmore 1998). Female blue tit song could play a role in intrasexual competition over mates and territories and to actively reduce the likelihood of polygyny, which often results in lower reproductive success for females (Schlicht and Kempenaers 2021). This could be in line with early reports of female blue tit song during “reproductive fighting” (Hinde 1952), but this working hypothesis must be tested by future experimental work, measuring whether females sing or react differently to the presentation of simulated male and female intruders.

Next to a potential role in territorial defense, female solo song may also be involved in mate choice and pair formation (Langmore and Davies 1997; Langmore 1998). Female blue tits displayed solo song during daytime singing from winter to spring (as early as 17 of January, the earliest sampling date in our record). Solo singing in winter is of special interest as the social associations during this time are related to the formation of breeding pairs (Beck et al. 2020). The early pair formation in blue tits also raises questions as to why males, but not females, sing prolifically at dawn during the fertile period. In blue tits, dawn song is tightly associated with seeking within- and extra-pair copulations by males (Welling et al. 1995; Kempenaers et al. 1997; Poesel et al. 2004; Parker et al. 2006), and with territory defense (Poesel et al. 2004). Typically, male dawn song ends when the female exits the nest and partners copulate (Sierro J, personal observation; Poesel et al. 2004; Parker et al. 2006). Normally, whether a copulation takes place or not is under female control (Kempenaers et al. 1995) and this, with the presence of frequent extra-pair copulations (Leech et al. 2001), implies a particular selection pressure on male song that appears absent in female song. Given such a contextual difference in singing behavior around the time of copulation, we can infer that selection pressures shaping song are not the same between the sexes (Austin et al. 2021), even though we need to know more about the function of female song in blue tits. Theoretically, such differential pressures could imply differences in song parameters that play a role in female choice during extra-pair copulations (Kempenaers et al. 1997; Austin et al. 2021), which is consistent with our results.

Indeed, we found that certain song traits differed between sexes, but we also found age and seasonal correlates with song that were similar in both sexes. Peak frequency was negatively correlated with age in both females and males. Female song, like male song, also varied seasonally in acoustic structure, decreasing in peak frequency, and increasing in vocal consistency towards the fertile period. These results provide female song with communicative value in many contexts since age is an important factor associated with contest success or reproductive capacity (Bradbury and Vehrencamp 1998). Furthermore, we show circumstantial evidence of spectral flexibility within individuals and song types, indicating that individuals may shift their songs to lower frequencies during the fertile period. This contrasts with previous reports of lower song frequencies during the fertile period in a related species, the great tit, that were attributed to changes in the use of song types rather than a frequency shift of the same song type (Halfwerk et al. 2011). To our knowledge, this is the first study to document seasonal changes in acoustic structure of female song, which could be of relevance in communication during different breeding stages. In males, such seasonal variation has been associated with specific functions of song during reproduction (Ballentine et al. 2003; Halfwerk et al. 2011; Vehrencamp et al. 2013). Future studies that shed light into the functional role of female song may help us to understand our observed variation in song traits.

From this quantitative assessment of female song in blue tits, we can conclude that female blue tits sing throughout the breeding season albeit with a lower total song output than males. The behavioral contexts of female song overlap with those in males, but females do not produce dawn song. Female song traits correlate with age and show significant seasonal variation indicating that variation in female song could have communicative value and may play a role in inter- or intrasexual interactions. Future experimental work should address which sexual differences and similarities in song are meaningful in communication and which selection pressures shaped the observed differences. Finally, increasing documentation of female secondary sexual traits is crucial for our understanding of the evolution of sexual signals in animal communication and should inspire large-scale comparative studies of female sexual signaling.

## Supplementary Material

arac044_suppl_Supplementary_Figure_S1

arac044_suppl_Supplementary_Figure_S2

arac044_suppl_Supplementary_Figure_S3

arac044_suppl_Supplementary_Figure_S4

## Notes

We want to thank Dr. Thomas Mondain-Monval for his helpful advice on various aspects of the statistical analysis. Moreover, we are grateful to Dr. Stuart Sharp and Dr. Sarah Collins for their comments on an earlier version of the manuscript. Finally, we also thank the referees that revised this manuscript during the peer-reviewed process for their contributions.

Data availability: Analyses reported in this article can be reproduced using the data provided by Sierro et al. (2022).
