# Supplementary figures and images for: Female blue tits sing frequently: a sex comparison of occurrence, context, and structure of song
Source: Behav Ecol. 2022 Jun 20;33(5):912–25. doi: 10.1093/beheco/arac044 (PMC9639586; doi:10.1093/beheco/arac044)

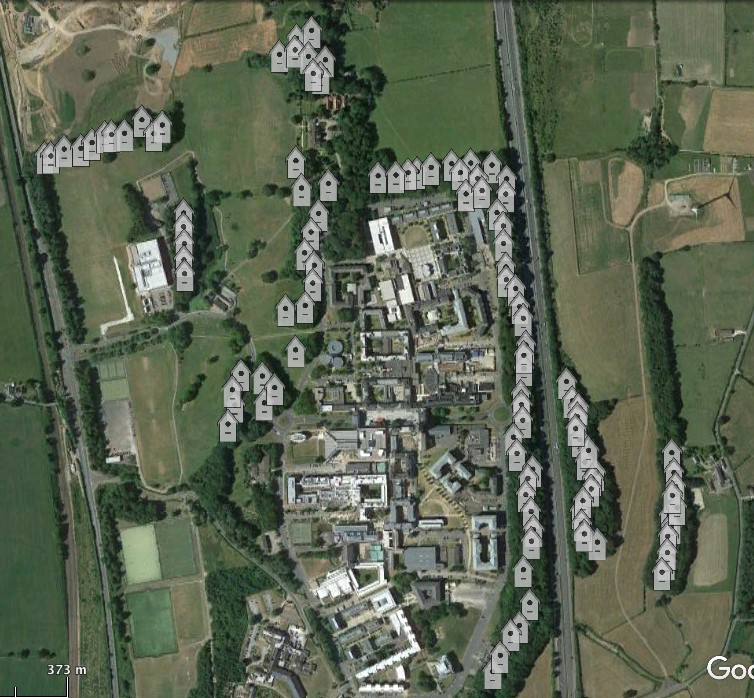

Supplement: arac044_suppl_Supplementary_Figure_S1 [file arac044_suppl_supplementary_figure_s1.jpeg]

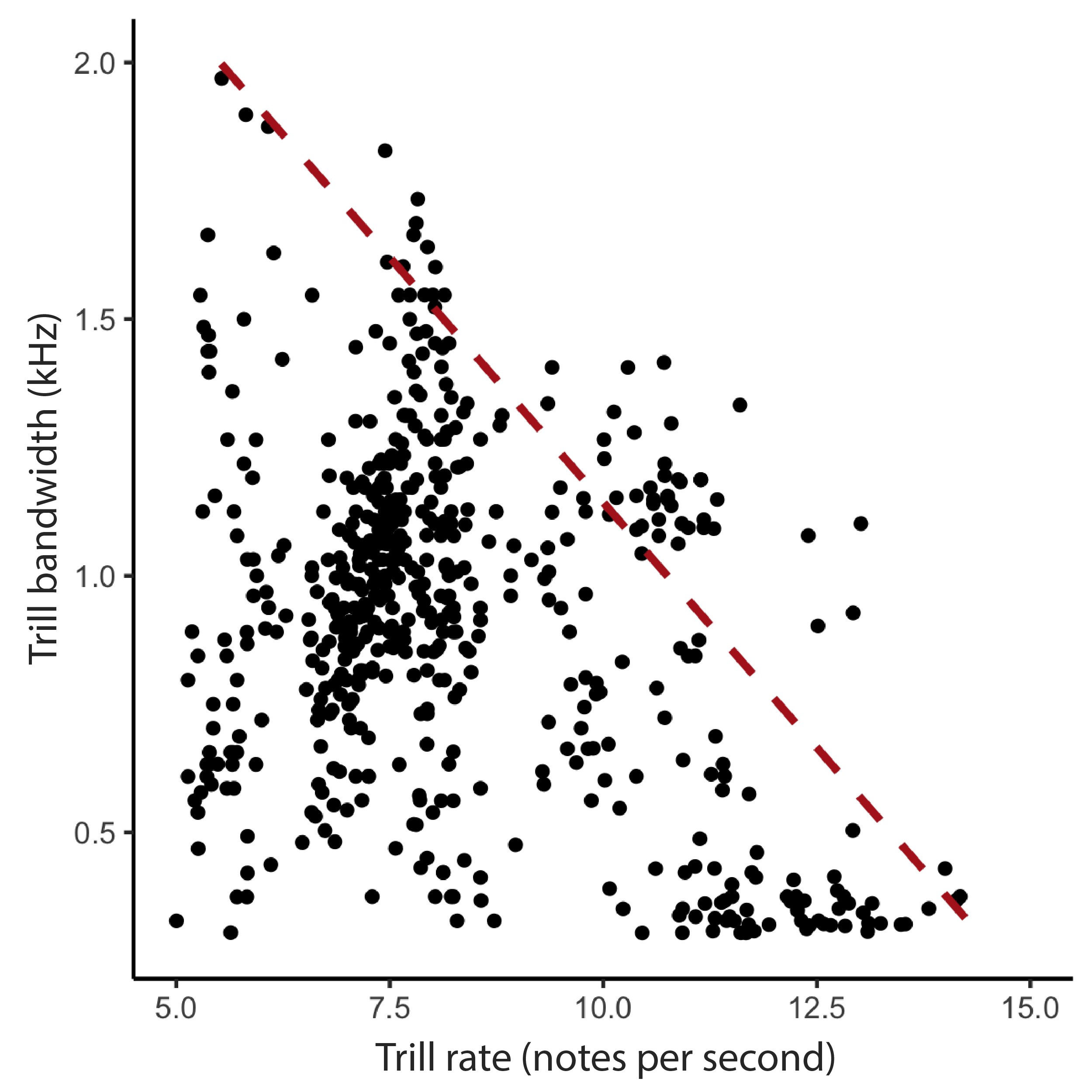

Supplement: arac044_suppl_Supplementary_Figure_S2 [file arac044_suppl_supplementary_figure_s2.jpeg]

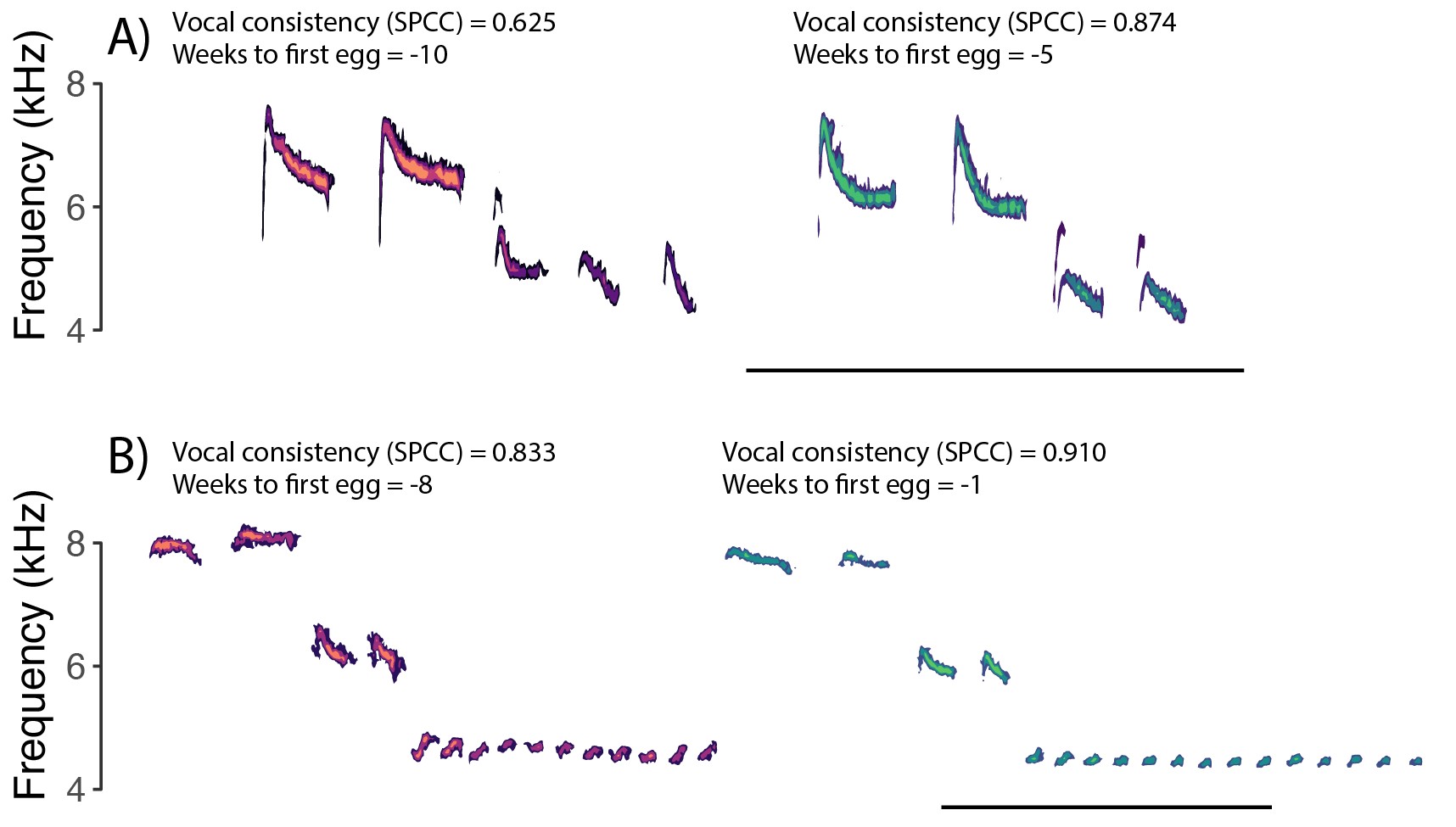

Supplement: arac044_suppl_Supplementary_Figure_S4 [file arac044_suppl_supplementary_figure_s4.jpeg]
