# Supplementary material for: Female blue tits sing frequently: a sex comparison of occurrence, context, and structure of song
Source: Behav Ecol. 2022 Jun 20;33(5):912–25. doi: 10.1093/beheco/arac044 (PMC9639586; doi:10.1093/beheco/arac044)

Figure S3

BRW3OM

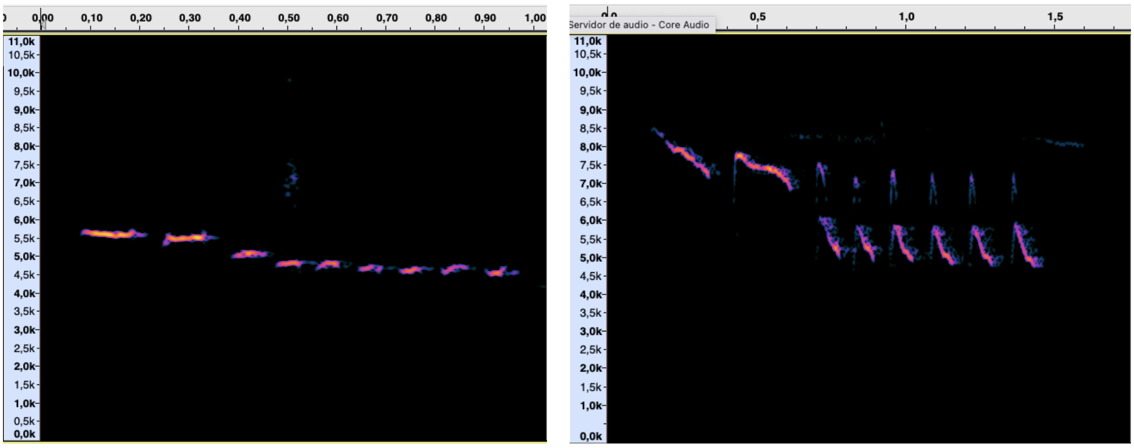

LGRM

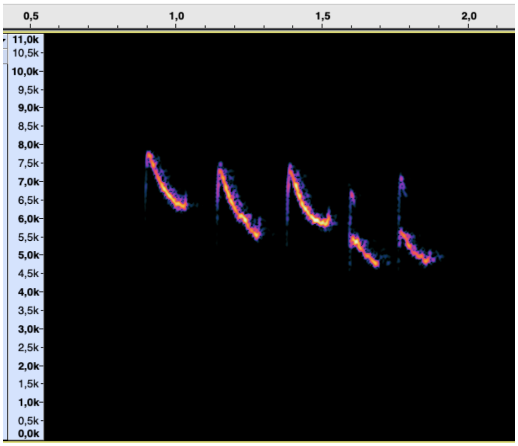

BYLM

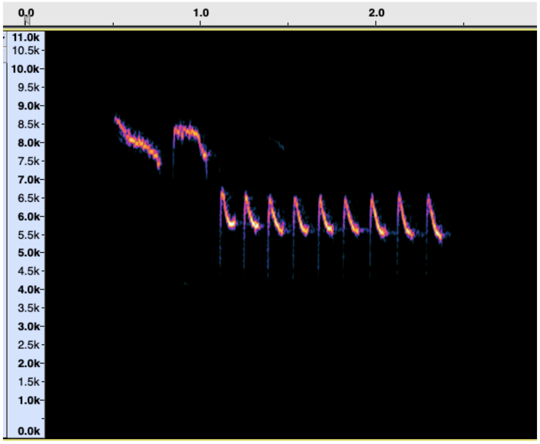

GMLG

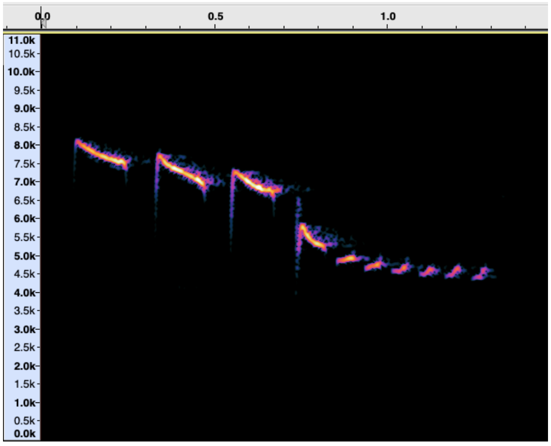

GYRW3M

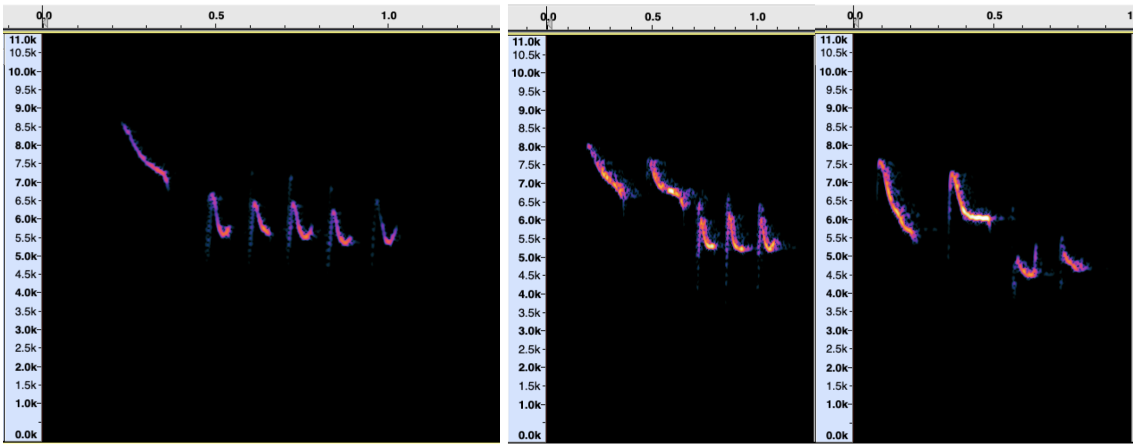

YBYM

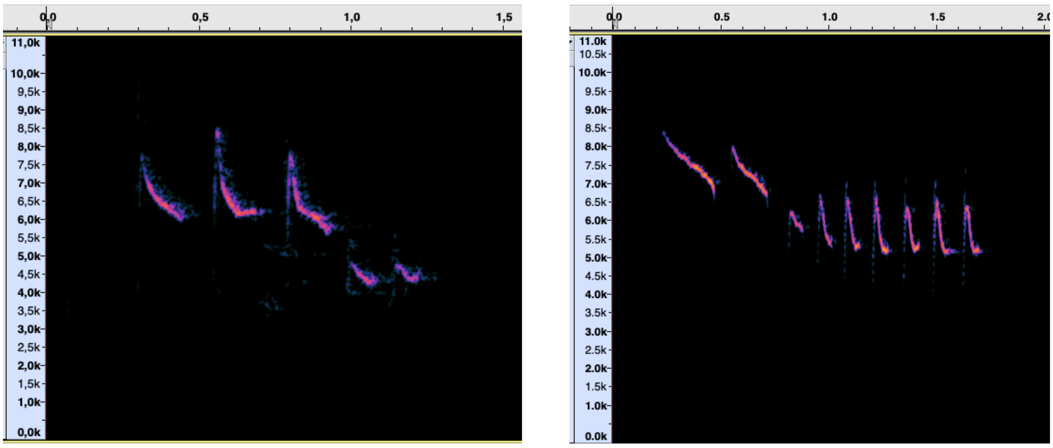

BBWM

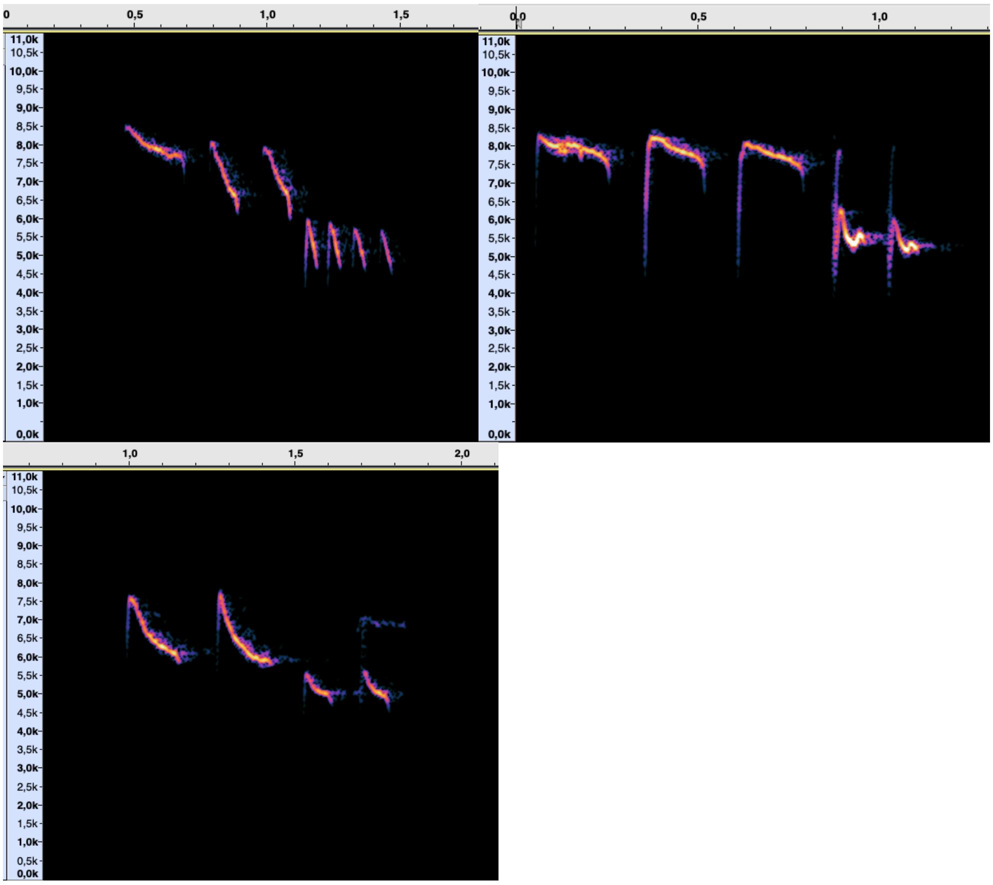
0 0,5 1,0 1,5
0,0 0,5 1,0
1,0 1,5 2,0
11,0k 10,5k 10,0k 9,5k 9,0k 8,5k 8,0k 7,5k 7,0k 6,5k 6,0k 5,5k 5,0k 4,5k 4,0k 3,5k 3,0k 2,5k 2,0k 1,5k 1,0k 0,5k 0,0k

GBNM

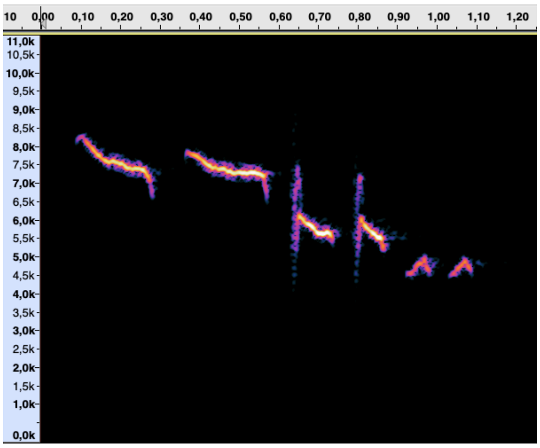
10 0,00 0,10 0,20 0,30 0,40 0,50 0,60 0,70 0,80 0,90 1,00 1,10 1,20
11,0k 10,5k 10,0k 9,5k 9,0k 8,5k 8,0k 7,5k 7,0k 6,5k 6,0k 5,5k 5,0k 4,5k 4,0k 3,5k 3,0k 2,5k 2,0k 1,5k 1,0k 0,0k

BGOM

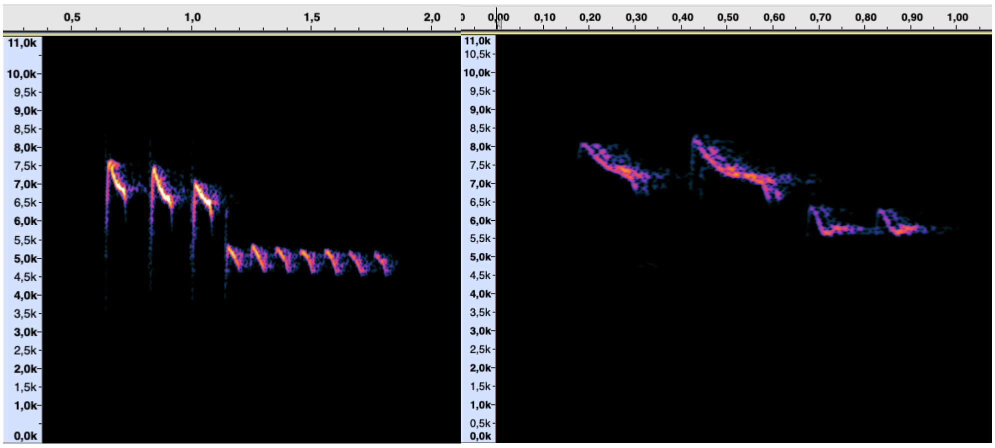

GMOB

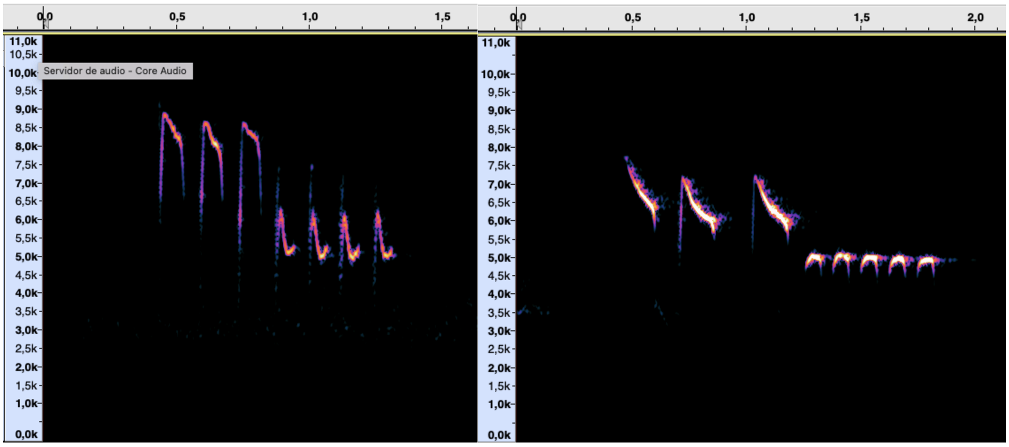

LMGL

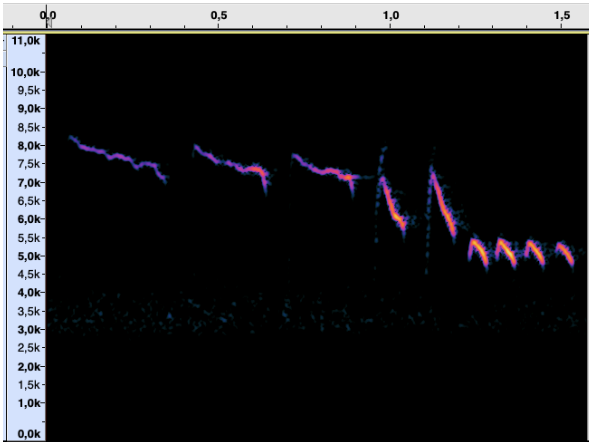

YMBR

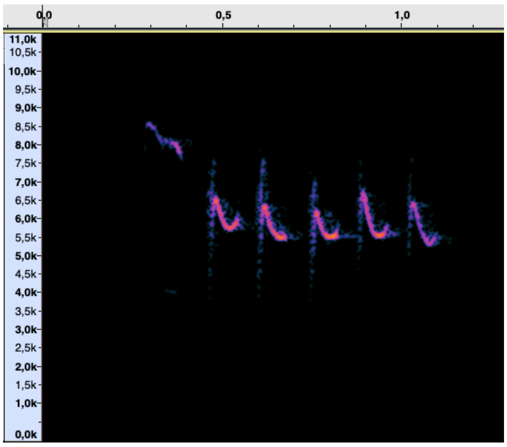

BYVM

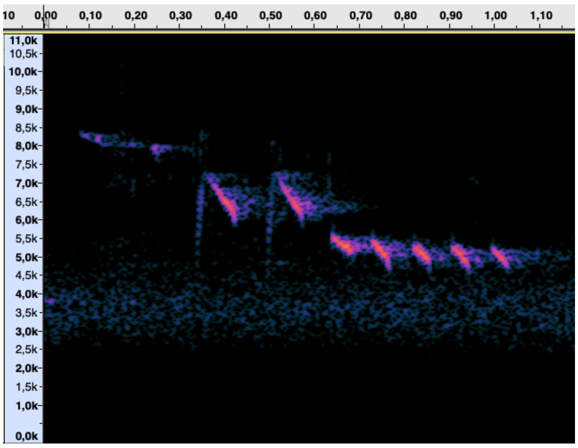

PMVV

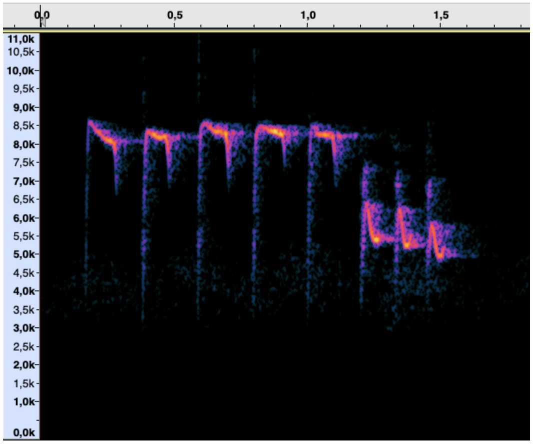

RMRW3V

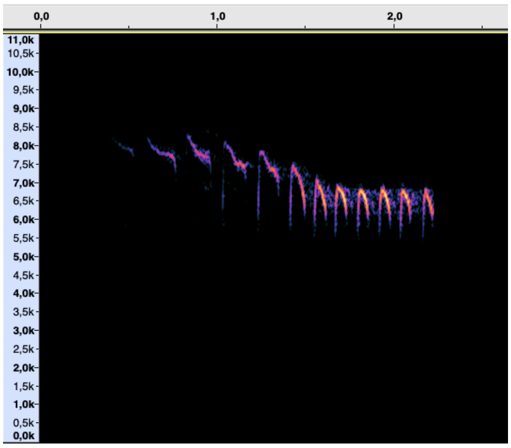

GYPM

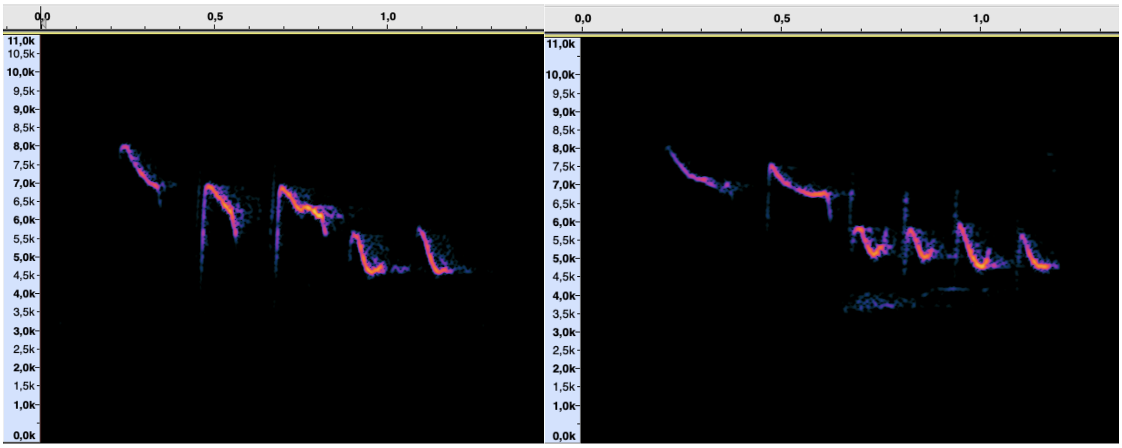

LMBR

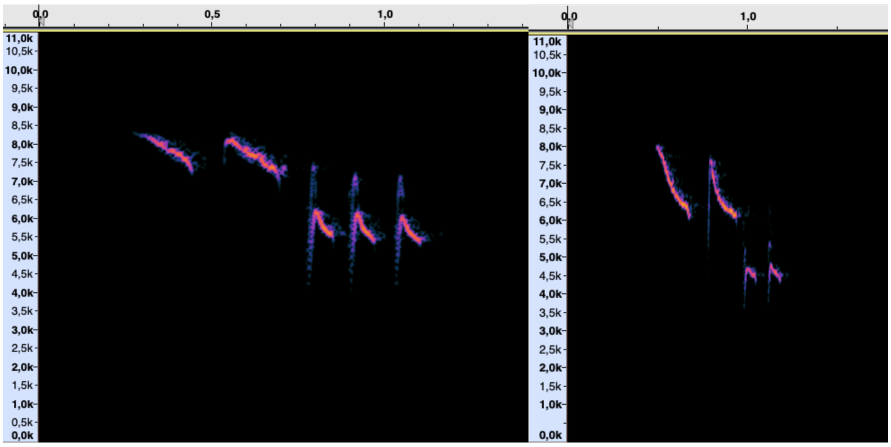

VMGB

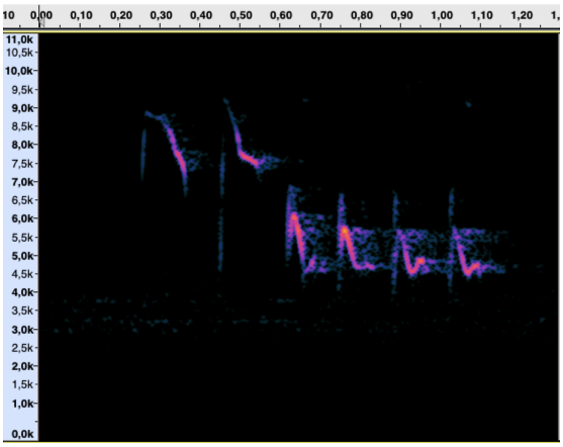

BPNM

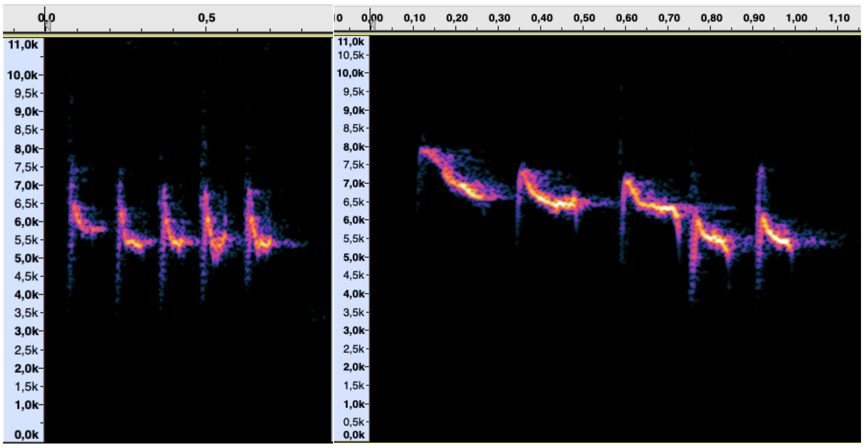

YYGM

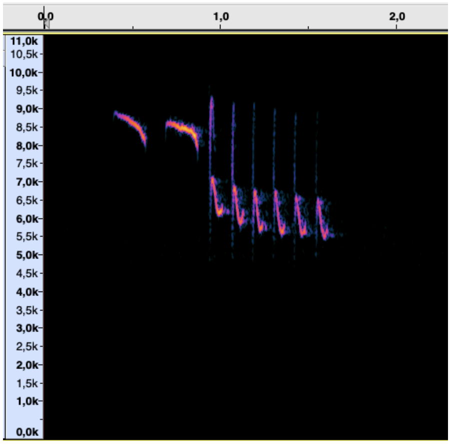

GMBG

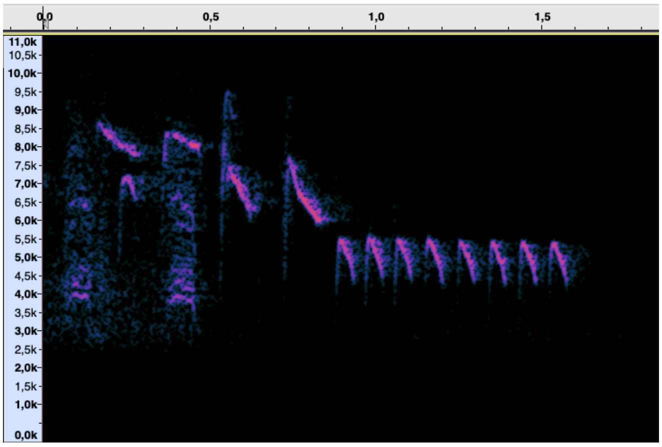

LGPM

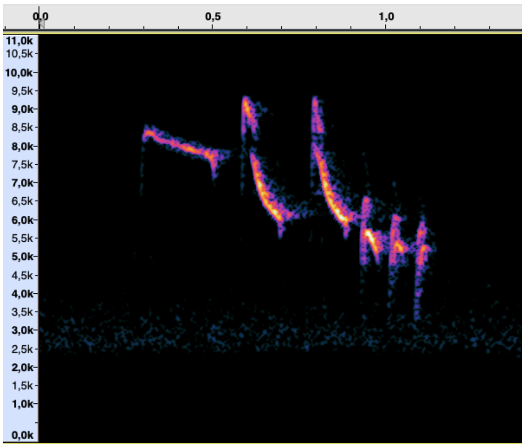

OMRG

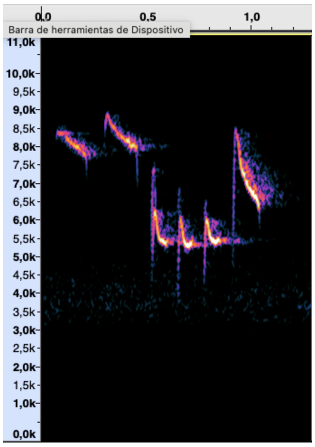

WBRM

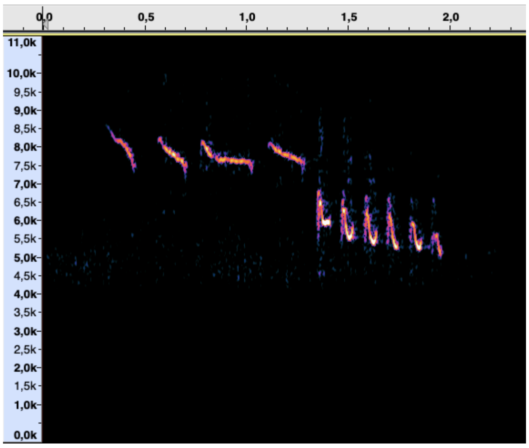

WMBW

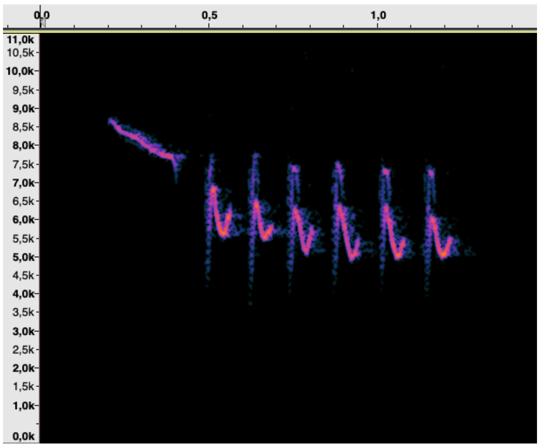

YMGB

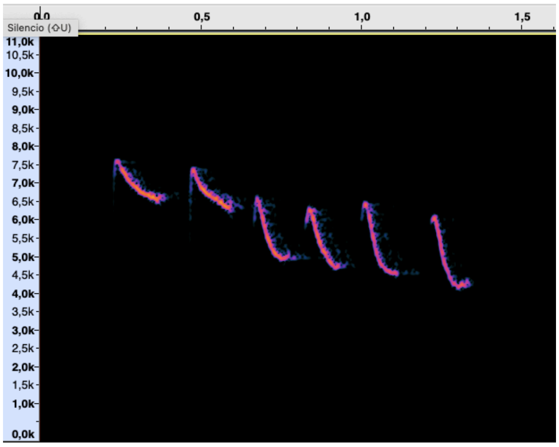

BRW3VM

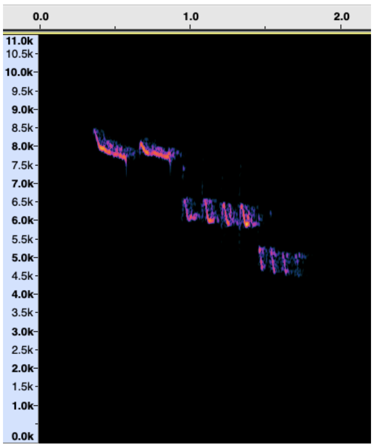

GMVY

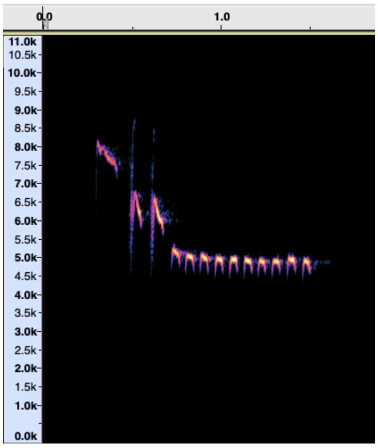

LMRW3G

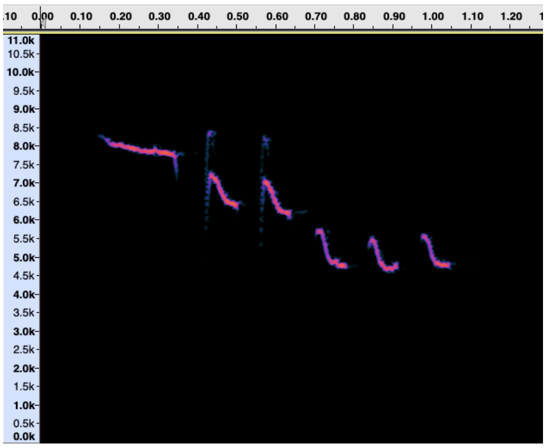

RLGM

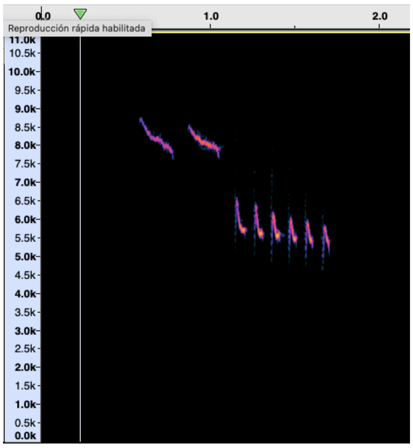

WMBL

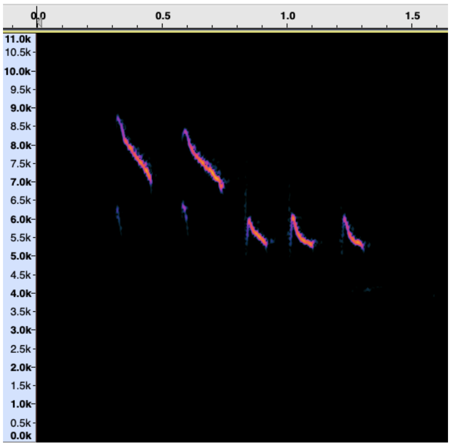

RPGM

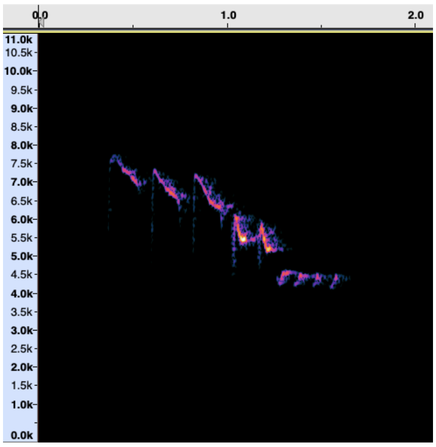

LMWB

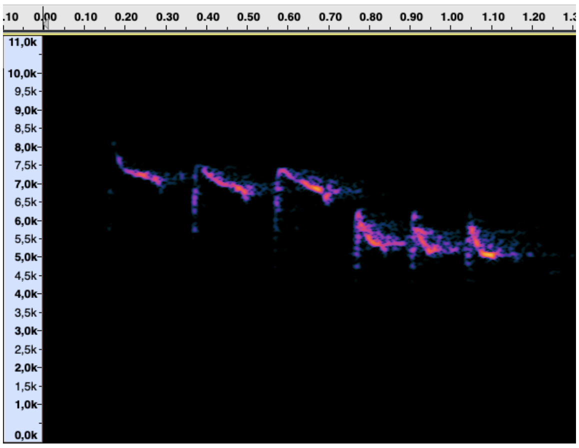

OMBY

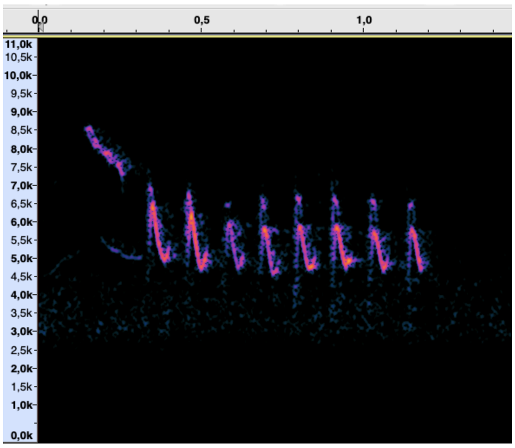

WMYG

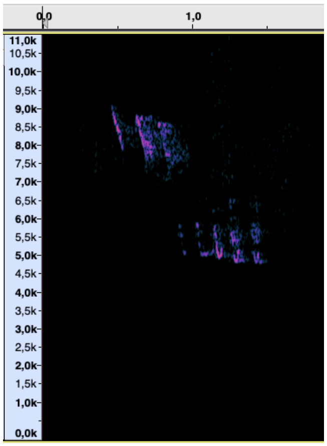

Supplement: arac044_suppl_Supplementary_Figure_S3 [file arac044_suppl_supplementary_figure_s3.pdf]
